# Supplementary material for: Assessing the Potential Distribution of Oxalis latifolia, a Rapidly Spreading Weed, in East Asia under Global Climate Change
Source: Plants (Basel). 2023 Sep 13;12(18):3254. doi: 10.3390/plants12183254 (PMC10537521; doi:10.3390/plants12183254)
Supplement: Supplementary file 1 [file plants-12-03254-s001.zip › Figure S1.pdf]

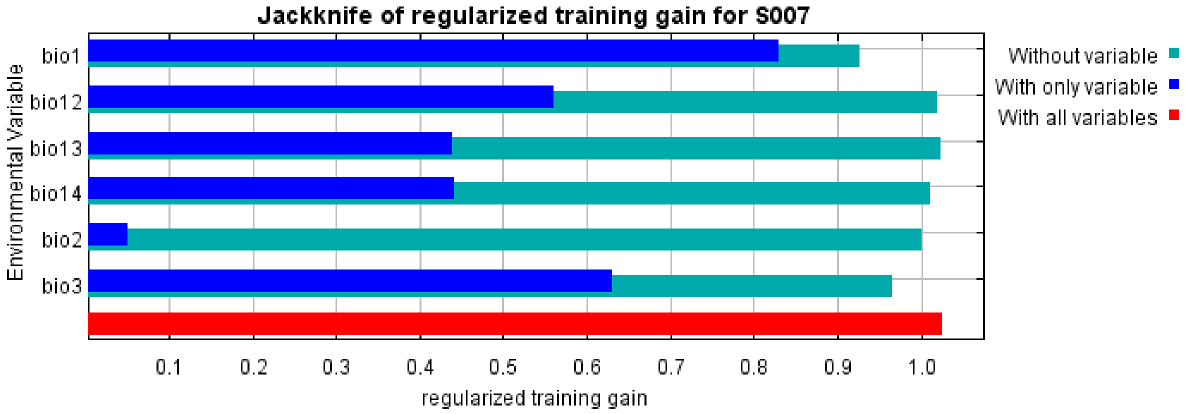

Figure S1a. Jackknife test analysis for evaluating test performance of *O. latifolia* under the current climate (1973–2000).

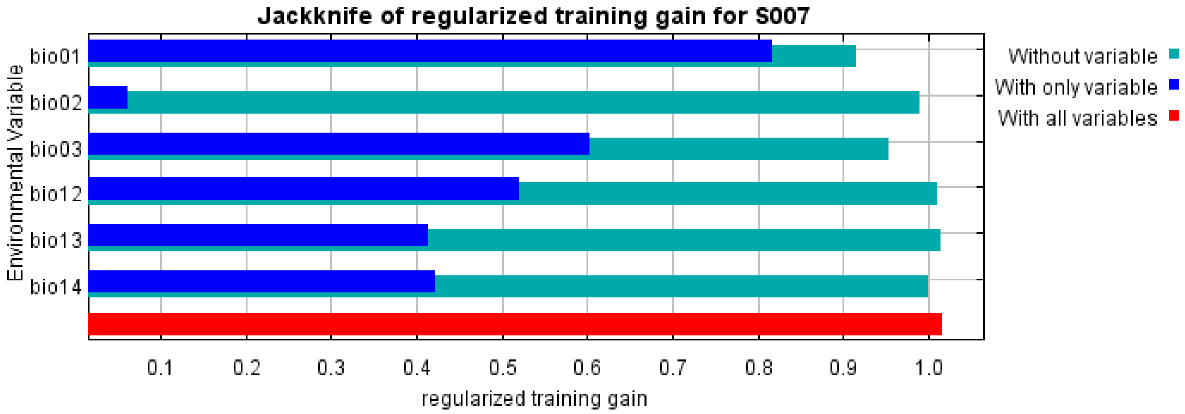

Figure S1b. Jackknife test analysis for evaluating test performance of *O. latifolia* under the climate change scenario SSP2-4.5 for the time period 2041–2060.

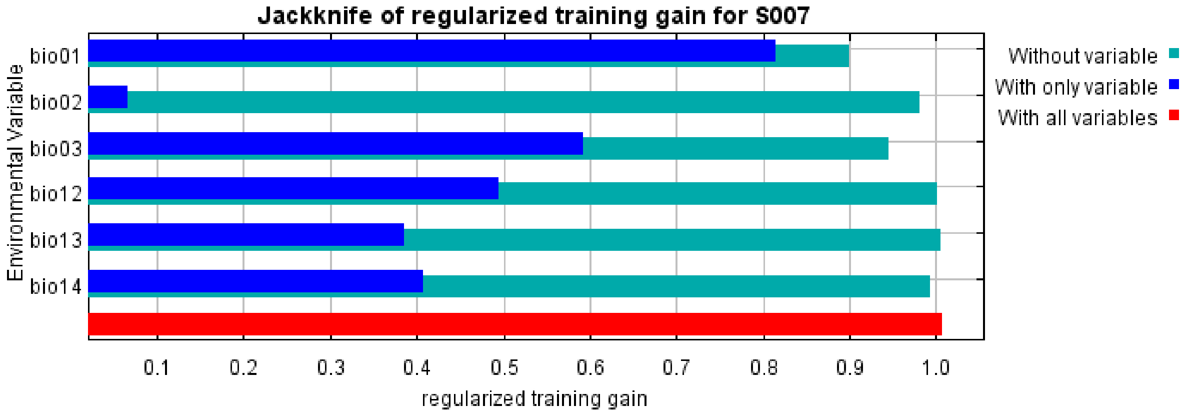

Figure S1c. Jackknife test analysis for evaluating test performance of *Oxalis latifolia* under the climate change scenario SSP2-4.5 for the time period 2081–2100.

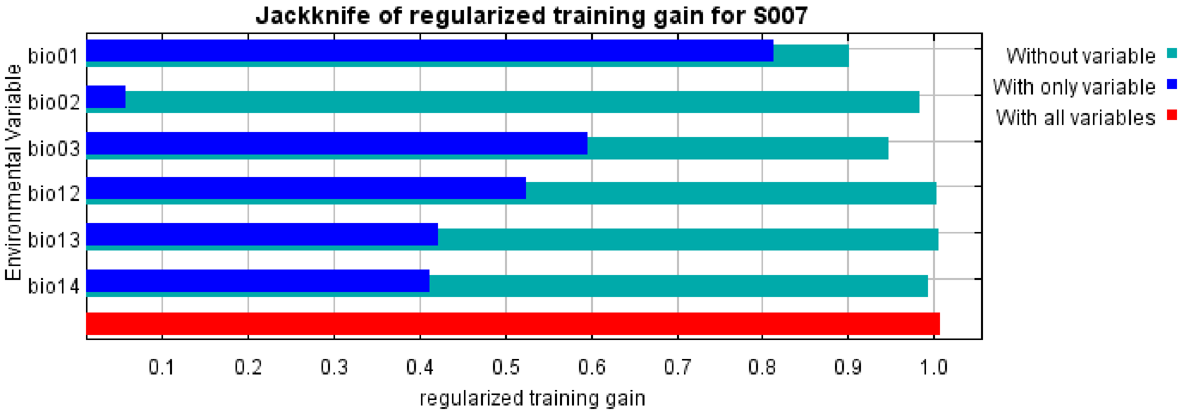

Figure S1d. Jackknife test analysis for evaluating test performance of *O. latifolia* under the climate change scenario SSP5-8.5 for the time period 2041–2060.

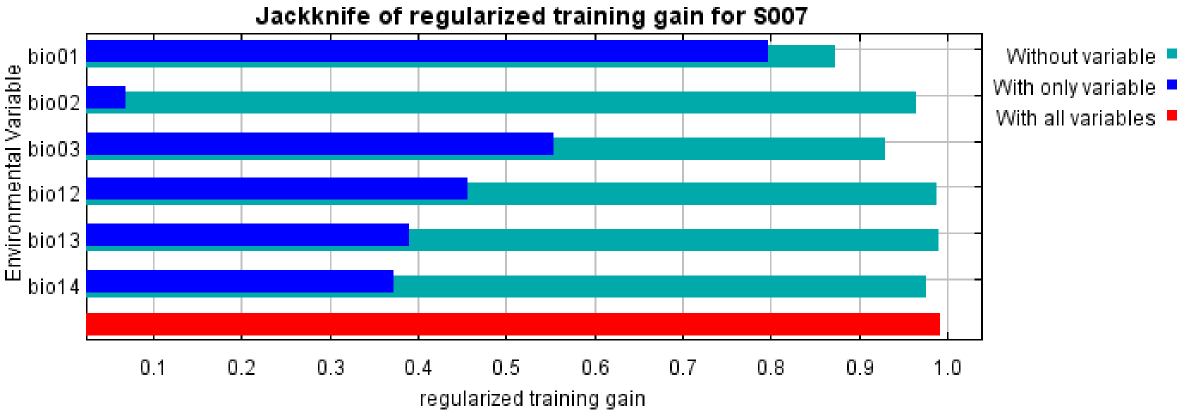

Figure S1e. Jackknife test analysis for evaluating test performance of *O. latifolia* under the climate change scenario SSP5-8.5 for the time period 2081–2100.
